# The Effects of Chronic Cigarette Smoking on Gray Matter Volume: Influence of Sex

**DOI:** 10.1371/journal.pone.0104102

**Published:** 2014-08-04

**Authors:** Teresa R. Franklin, Reagan R. Wetherill, Kanchana Jagannathan, Barbara Johnson, Joel Mumma, Nathan Hager, Hengyi Rao, Anna Rose Childress

**Affiliations:** 1 Perelman School of Medicine at the University of Pennsylvania, Department of Psychiatry, Philadelphia, Pennsylvania, United States of America; 2 Perelman School of Medicine at the University of Pennsylvania, Center for Functional Neuroimaging, Philadelphia, Pennsylvania, United States of America; Hangzhou Normal University, China

## Abstract

Cigarette smoke contains nicotine and toxic chemicals and may cause significant neurochemical and anatomical brain changes. Voxel-based morphometry studies have examined the effects of smoking on the brain by comparing gray matter volume (GMV) in nicotine dependent individuals (NDs) to nonsmoking individuals with inconsistent results. Although sex differences in neural and behavioral features of nicotine dependence are reported, sex differences in regional GMV remain unknown. The current study examined sex differences in GMV in a large sample of 80 NDs (41 males) and 80 healthy controls (41 males) using voxel-based morphometry. Within NDs, we explored whether GMV was correlated with measures of cigarette use and nicotine dependence. High-resolution T1 structural scans were obtained from all participants. Segmentation and registration were performed in SPM8 using the optimized DARTEL approach. Covariates included age and an estimate of total global GMV. Differences were considered significant at *p*≤0.001, with a whole brain FWE-corrected cluster probability of *p*<0.025. Among NDs compared to Controls less GMV was observed in the thalamus and bilateral cerebellum and greater GMV was observed in the bilateral putamen and right parahippocampus. Lower thalamic GMV was observed in both female and male NDs compared to Controls. Female NDs also had lower GMV in the left cerebellum and in the ventral medial and orbitofrontal cortices with no areas of greater GMV. Male NDs had lower GMV in bilateral cerebellum and greater GMV in bilateral parahippocampus and left putamen. Within male NDs, GMV in the left putamen was correlated with number of pack years. This study, conducted in a large cohort, contributes to our knowledge of brain morphology in nicotine addiction and provides additional evidence of sex-specific effects on GMV in NDs. Identifying brain vulnerabilities with respect to sex provides a methodological framework for personalized therapies to improve relapse rates for both sexes.

## Introduction

Cigarette smoking is the leading cause of preventable morbidity and premature mortality worldwide [1]. Although most nicotine dependent individuals (NDs) express the desire to quit smoking, relapse rates remain high [2], [3]. Preclinical studies indicate that chronic exposure to nicotine, the addictive component in cigarettes, causes significant neurochemical and pathological brain changes in fetal and adolescent rats [4], [5], [6]. In addition to nicotine’s potential harmful effects, cigarette smoke contains numerous toxic chemicals that could contribute to the significant adverse health consequences in individuals who are chronic long-term cigarette smokers, including anatomical and chemical brain changes [7], [8], [9]. Understanding the vulnerabilities of the nicotine-dependent brain will aid in the development of successful treatment strategies to help us conquer this devastating and difficult to treat addiction.

Voxel-based morphometry (VBM) studies have examined the effects of chronic smoking on the brain by comparing gray matter volume (GMV) in otherwise healthy nicotine dependent individuals (NDs) to nonsmoking individuals (Controls) with inconsistent results [10], [11], [12], [13], [14], [15], [16]. Inconsistencies may be related to differences in methodologies and/or the populations studied. For example, although some studies identified the thalamus as a region of lower GMV in NDs [11], [12], this was not observed in other studies [13], [14], [15], [17]. Further, not all studies showed correlations with relevant smoking-related behavior. Specifically, Gallinat et al. (2006) reported that greater number of pack-years were associated with less GMV in frontal and temporal lobes; whereas, Zhang et al. (2011a) found an association between greater number of pack-years and less GMV in the left prefrontal cortex, but only in high pack-year NDs [14]. These studies, and others, suggest structural abnormalities in ND that may be related to lifetime cigarette exposure and that additional study in larger cohorts is necessary.

Although previous research highlights the adverse effects of cigarette smoking on neural structure, sex differences were not examined. Sex-specific patterns of regional GMV differences among smokers and between smokers and healthy individuals might be expected, given that numerous studies report sex differences in regional cortical and subcortical volumes in various brain disorders [18], [19] including drug and alcohol dependence [20], [21]. Preclinical and clinical studies indicate that females are more vulnerable to cigarette smoking initiation, nicotine dependence and have more difficulty quitting than males [22], [23]. Although the exact mechanisms underlying these sex differences remain unknown, research suggests that sex differences in neural functioning and organization may influence aspects of nicotine dependence [24], [25]. Knowledge of sex-specific neurophysiological anomalies in GMV associated with nicotine and cigarette smoking may provide a methodological framework for personalized therapies to improve relapse rates.

To this end, the goal of the current study was to examine differences in GMV and sex differences in GMV between NDs and matched Controls. Based on the known sex-specific differences in brain function and nicotine dependence, we hypothesized that male and female smokers would show different patterns of GMV alterations. Within ND individuals, we explored whether regional GMV was associated with cigarette use and nicotine dependence. The VBM tool in Statistical Parametric Mapping software (SPM8; Wellcome Department of Cognitive Neurology; http://www.fil.ion.ucl.ac.uk/spm) was used to accomplish this goal.

## Methods

### 1. Subjects

Physically healthy NDs and Controls were recruited via advertisements, flyers, and referrals for fMRI studies at the University of Pennsylvania. After completing an initial telephone screen, individuals were given a description of their respective study and written informed consent was obtained. Screening procedures included a history and physical examination and a psychological assessment. The Fagerstrom Test for Nicotine Dependence (FTND) [26] assessed severity of nicotine dependence among NDs. Exclusion criteria included current DSM-IV Axis I diagnoses (other than nicotine dependence in smokers), lifetime history of head injury with loss of consciousness for more than 3 minutes, significant alcohol or drug history, contraindications for MRI, and clinically significant medical conditions. Urine drug screens verified the absence of illicit drugs in all subjects and the absence of nicotine and its major metabolite, cotinine, in Controls. Eighty NDs (41 males) and 80 Controls (41 males) matched by age, sex, and education years, met inclusion/exclusion criteria (Table 1). The study adhered to the Declaration of Helsinki and was approved by the University of Pennsylvania Institutional Review Board.

**Table 1:** Characteristics of nicotine-dependent cigarette smokers and non-smoking controls.

	NDs (n = 80)				Controls (n = 80)			
	*Male*		*Female*		*Male*		*Female*	
	n	%	n	%	n	%	n	%
	41	51.3	39	48.7	41	51.3	39	48.7
	*Mean*	*SD*	*Mean*	*SD*	*Mean*	*SD*	*Mean*	*SD*
Age (years)	35.7	11.1	31.9	10.6	33.2	7.5	30.9	7.2
Education level	14.1	2.0	14.8	2.4	13.6	2.2	14.2	2.0
Years smoking	16.0	11.3	12.0	8.4	–	–	–	–
CPD^a^	16.1	6.1	13.2	5.6	–	–	–	–
FTND	4.6	1.7	4.3	1.9	–	–	–	–
Pack years^b^	13.0	9.9	7.9	6.0	–	–	–	–

SD, standard deviation; CPD, cigarettes smoked per day; FTND, Fagerstrom Test for Nicotine Dependence; Pack-years, smoking years X CPD/20.

a Male NDs>female NDs, *t*(79) = 2.21, *p* = 0.03.

b Male NDs>female NDs, *t*(79) = 2.81, *p* = 0.007.

### 2. MR Acquisition

NDs smoked a cigarette to satiety approximately 45 minutes prior to scan acquisition. Imaging data were acquired on a Siemens 3 Tesla Trio whole-body scanner (Erlangen, Germany) at the Hospital of the University of Pennsylvania using a product eight-channel array coil. High-resolution T1-weighted anatomical images were obtained using a three dimensional- magnetization prepared rapid acquisition gradient echo (3D-MPRAGE) sequence (TR = 1620 ms, TE = 3 ms, flip angle = 15°, 160 contiguous slices of 1.0 mm).

### 3. Data Processing

Data were preprocessed using statistical mapping software, version 8 (SPM8; Wellcome Department of Cognitive Neurology, London, UK; www.fil.ion.ucl.ac/spm) implemented in MatlabR2013 (MathWorksInc., Natick, MA, USA). Each subject’s MR images were checked for structural abnormalities and reoriented to the anterior-posterior commissure line. Reorientated images were segmented into gray matter, white matter and cerebral spinal fluid in native space using the New Segmentation algorithm implemented in SPM8. This iterative procedure results in more accurate tissue classification. The resulting rigidly aligned and resliced gray and white matter tissue maps were imported into DARTEL (Diffeomorphic Anatomical Registration using Exponentiated lie Algebra), an advanced high-dimensional diffeomorphic registration algorithm within SPM8 that has been shown to improve spatial precision [27], [28]. Subsequently, the study-specific gray matter templates were created from the imported maps of all subjects. The final average template was generated after six iterations by averaging all of the aligned images. During successive iterations, flow fields for each subject’s images were yielded to parameterized deformations by warping to the template and then these were employed in the modulation step. The gray matter map of each subject was transformed to Montreal Neurological Institute (MNI) space by applying the deformation parameters. Next, Jacobian modulation was applied to the warped images of gray matter. The normalized modulated images were then smoothed with an 8-mm full width half maximum isotropic Gaussian kernel for statistical analyses. Data were visually inspected following each pre-processing step to ensure integrity.

### 4. Data analysis

Statistical parametric maps were created in SPM8 to perform between-group comparisons using the gray matter tissue segmentation output by DARTEL. A 2×2 full factorial model was used to explore differences in GMV between NDs and Controls, NDs and Controls by sex, and group X sex interactions. As it has been demonstrated that both age and total global brain measures yield significant effects on regional GMV [29], [30], covariates included age and an estimate of total global GMV. Whole-brain GMV analyses were conducted using random field-based cluster-size testing, with structural differences considered significant if *p*≤0.001, with a whole brain FWE-corrected cluster probability of *p*<0.025. GMV in clusters that differed between NDs and Controls were extracted using the MarsBar region of interest toolbox (http://marsbar.sourceforge.net/) and exported to IBM SPSS Statistics 19.0 (Armonk, NY) to investigate possible associations between GMV and cigarette use and dependence.

## Results

Table 1 provides subject characteristics. Analysis of variance (ANOVA) was used to compare subject characteristics across groups. There were no significant differences between NDs and Controls on age, sex, or education years. Consistent with previously reported sex-specific differences in smoking behavior [31], [32], [33], male NDs smoked a greater number of cigarettes per day and had higher pack-years than their female counterparts. Total global GMV was not different between NDs and Controls (591.50 mm^3^, SD 68.56 and 603.67 mm^3^, SD 73.58, *p = *0.27). As expected, total global GMV was greater in male NDs (629.02 mm^3^, SD 60.03) compared to female NDs (553.96 mm^3^, SD 55.01; *p*<0.001) and male Controls (639.11 mm^3^, SD 68.62) compared to female Controls (561.87 mm^3^, SD 52.64; *p*<0.001).

**Figure 1:**
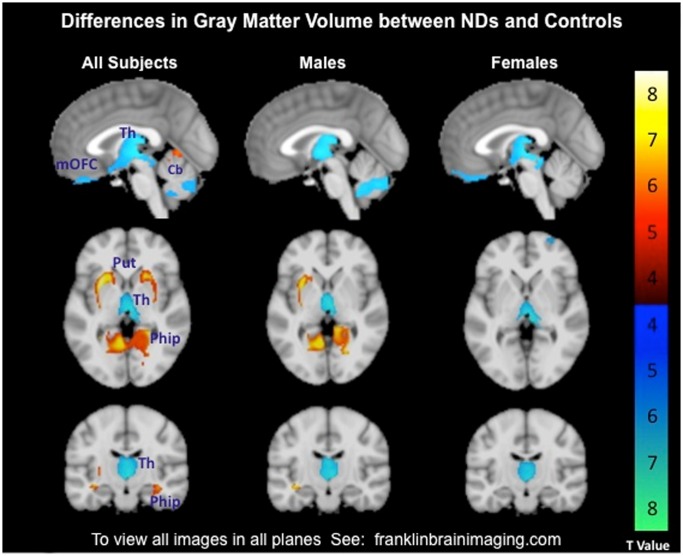
Differences in gray matter volume (GMV) between nicotine dependent (NDs; n = 80) and control subjects (Controls; n = 80) shown in all subjects and separately by sex (NDs, n = 41 male; Controls, n = 41 male). Representative MRI sagittal, axial, and coronal brain slices analyzed in SPM8 and overlain on the Montreal Neurological Institute brain. Slice shown is centered within the thalamus (x, y, z; 0, −16, 0). Hot colors represent regions of greater GMV, while cool colors represent regions of less GMV in NDs compared to Controls. T values range from 4.42 to 8.06, significant in whole brain analysis at *p*<0.001, and FWE cluster-corrected *p*<0.025. Images are displayed neurologically. An interactive visual display of all brain data in all three planes can be found at http://franklinbrainimaging.com (Available upon publication).

Table 2 provides a full summary of GMV differences. Among NDs compared to Controls, less GMV was observed in the thalamus, extending through to the medial orbitofrontal cortex, and bilateral cerebellum whereas greater GMV was observed in bilateral putamen and right parahippocampus (see Figure 1 and http://franklinbrainimaging.com: Available upon publication). There were sex-specific volume differences between NDs and Controls. Female NDs had less GMV than female Controls in the thalamus, left cerebellum, left ventrolateral prefrontal cortex, and medial orbitofrontal cortex. There were no brain regions in which female NDs had greater GMV than female Controls. ND males had significantly less GMV in the thalamus and bilateral cerebellum and significantly greater GMV in the bilateral parahippocampus (extending into the left putamen) compared to male Controls. There were no significant group X sex interactions.

**Table 2:** Clusters of gray matter volume differences between NDs and Controls.

Region	Cluster size(voxels)	MNI coordinates			*T*-value	*p value* *FWE cor*
		*x*	*y*	*z*		
**Group effect**						
** NDs<Controls**						
Thalamus	1851	4	−18	2	8.06	0.001
Medial prefrontal cortex a		2	36	−30	4.78	
L Cerebellum	2827	−40	−70	−42	6.19	0.001
R Cerebellum	1987	30	−50	−50	4.83	0.001
** NDs>Controls**						
L Putamen	777	−28	8	−8	7.16	0.001
R Putamen	546	22	18	4	5.44	0.007
R Parahippocampus	2319	20	−40	−8	6.32	0.001
**Sex effect**						
** Females: NDs<Controls**						
Thalamus	891	6	−26	−2	7.07	0.001
L Cerebellum	943	−40	−72	−42	4.80	0.001
Ventral medial prefrontal cortex	540	36	60	−10	4.48	0.007
Medial orbitofrontal cortex	857	−6	24	−28	4.42	0.001
** Females: NDs>Controls**						
No significant clusters						
** Males: NDs<Controls**						
Thalamus	777	4	−14	0	5.45	0.001
R Cerebellum	1909	28	−54	−50	5.15	0.001
L Cerebellum	1627	−32	−64	−52	4.60	0.006
** Males: NDs>Controls**						
R Parahippocampus	660	18	−38	−8	6.30	0.001
L Parahipppocampus	472	−34	−10	−14	5.28	0.012
L Putamen^b^		−30	10	−2	5.13	

All clusters are significant in whole brain analysis at *p*<0.001, and FWE cluster-corrected *p*<0.025.

Coordinates listed are in Montreal Neurological Institute (MNI) space. L, left; R, right.

a extended from within the thalamic cluster.

b extended from within the left parahippocampal cluster.

Within the group of ND individuals, correlation analyses revealed that number of pack years was positively correlated with GMV in the left putamen in males (*r = *.38, *p* = 0.018). Additionally, GMV in the left cerebellum approached a significant inverse correlation with number of pack years in males (*r* = -.31, *p = *0.054). No significant associations were found between GMV and cigarettes per day and FTND among all NDs or among male or female NDs.

## Discussion

The purpose of this study was two-fold. Given the impact that chronic cigarette smoking may have on brain structure, the first goal was to conduct a systematic GMV study in a large cohort of NDs compared to Controls using an advanced registration method that provides greater sensitivity than traditional VBM. Additionally, given the importance of the influence of sex on brain structure and function in a variety of disorders, a second goal was to characterize sex differences in GMV in NDs, extending our knowledge of brain morphology in nicotine addiction. Among NDs compared to Controls, less GMV was observed in the thalamus, extending through to the medial orbitofrontal cortex (mOFC) and bilateral cerebellum whereas greater GMV was observed in bilateral putamen and right parahippocampus. Less thalamic GMV was observed in both female and male NDs. Female NDs also had lower GMV in the left cerebellum and in the ventral medial cortex and mOFC with no areas of greater GMV. Male NDs had lower GMV in bilateral cerebellum and greater GMV in bilateral parahippocampus extending into the left putamen. Positive associations were observed between pack years and GMV in the left putamen in male NDs. Additionally, GMV in the left cerebellum approached a significant inverse correlation with pack years in male NDs.

The strongest difference between NDs and Controls identified in the current report was observed in the thalamus wherein less GMV was detected in NDs as a group and in both male and female NDs. In humans, the thalamus has the highest density of nicotinic acetylcholine receptors of any brain region [34], [35], and thus, constant bombardment of these receptors by chronic cigarette smoking may make it a prime target for potential morphometric anomalies. Indeed, cigarette smoking has been shown to correlate with increased nicotine binding in the human thalamus [36]. Alternatively, and not mutually exclusive, this region may be particularly susceptible to one or more of the numerous carcinogens found in cigarette smoke (e.g., acetaldehyde, arsenic, benzene) [9]. The finding of less thalamic GMV is consistent with the findings of some studies [11], [12] but not all [10], [14], [15]. Given that cigarette smoking behavior is multi-faceted and complex, and that various aspects of the behavior are modulated by both known [37], [38], [39] and unknown factors, we speculate that differences in other characteristics across studies are contributing to the variable results.

The cerebellum was also identified as a region wherein less GMV was observed in NDs compared to Controls; however, sex-specific analyses show this finding to be stronger and bilateral in male NDs. Yu and colleagues (2011) studied only males and also identified a region of the cerebellum (bilateral) as a region of lower GMV in ND [13]. Kuhn and colleagues (2012) conducted a GMV study in NDs versus Controls specifically targeting the cerebellum [17]. The study, conducted in both sexes, showed significantly less GMV in the right cerebellum compared to Controls, with peak coordinates similar to those observed in the current study. Our results provoke speculation that the cerebellar effects observed by Kuhn et al. (2012) may be sex-specific. NDs show greater density of nicotine binding in the cerebellum (from 70–200%) compared to nonsmoking individuals [40], and brain blood flow in the cerebellum is increased by cigarette smoking [41], [42]. Further, plasma nicotine levels, which are elevated by smoking cigarettes, correlate with increased cerebellar blood flow [41]. Thus, potential pathophysiological effects associated with chronic smoking could at least partially account for this result. The effect may be stronger in males because of their greater exposure to cigarettes (i.e., greater number of pack-years).

The cluster of less thalamic GMV in NDs extended medially through the brain to the medial frontal gyrus, including the mOFC. GMV in prefrontal regions was unchanged in male NDs compared to male Controls but was less in female NDs compared to female Controls. Most previous studies have demonstrated reduced prefrontal GMV in NDs [10], [11], [12], [16] while some studies have not [14], [15]. Our results showing that this finding may be ‘carried’ by female NDs, may help explain these inconsistencies. Some support for this idea is provided by Yu and colleagues (2011), who studied only males, and did not find differences between NDs and Controls in prefrontal regions [13]. Given the role of the medial ventral regions of the prefrontal cortex in reward [43], [44] and the development and maintenance of addiction [45], [46], it is surprising that this finding was observed only in females, however resting cerebral blood flow (CBF) differs between male and female NDs in the mOFC [24], which could potentially lead to differences in regional GMV. Further, the mOFC is heavily involved in inhibition of behavior [47]. Less GMV in this region in female NDs may be related to the greater difficulty female NDs have in quitting smoking compared to male NDs [22].

In the present study, NDs had more GMV than Controls in the bilateral putamen. Striatal abnormalities are thought to underlie habitual, compulsive drug seeking and use [48], [49] and other psychiatric disorders characterized by repetitive, compulsive behaviors [50], [51]. Further, a meta-analysis has shown that severity of compulsivity has been associated with greater putamen GMV [52]. As such, the greater putamen GMV observed in NDs may be associated with long-term, compulsive cigarette smoking behavior. This suggestion is mildly supported by the finding of a positive association between pack years and GMV in the left putamen in male NDs.

In the present study, the parahippocampus showed greater GMV in NDs compared to Controls, but sex-specific analyses showed greater GMV in the parahippocampus only in male NDs. In direct comparisons between sexes we have shown that appetitive smoking cues (reminders to smoke) evoke greater bilateral hippocampal/amygdalar activation among males [24]. The hippocampus and amygdala are structures associated with emotional and drug memories [53], [54] and as such, our previous findings may be explained by sex-specific memory-related differences to smoking cues. Specifically, females may be more efficient at retrieving autobiographical memories [55], [56], while males show greater hippocampal activation to autobiographical memories [57]. Thus, males may require greater memory-related, hippocampal-amygdalar brain activity when retrieving cue-associated memories. Increased activity in brain is theoretically coupled to the delivery of life-sustaining blood and nutrients. One might tentatively speculate that chronic increased activity in this region, may lead to a relative expansion in GMV.

We hypothesized that the toxic chemicals found in tobacco smoke cause detrimental effects on brain morphology. Using pack-years as an estimate of lifetime cigarette smoke exposure, we explored whether associations existed between GMV and cigarette exposure. Associations between GMV and pack years were observed only in males; positive associations were observed in the putamen and negative associations were observed in the cerebellum. These results are inconsistent with the extant literature, which itself is inconsistent. Specifically, Gallinat et al. (2006) reported that greater pack-years were associated with less GMV in frontal and temporal lobes; whereas, Zhang et al. (2011a) found an association between greater pack-years and less GMV in the left prefrontal cortex, but only in high pack-year NDs [11], [14]. In a separate report, Zhang et al. (2011b) observed a trend in dorsolateral prefrontal cortex, yet no correlations were observed in the ROI analysis conducted by Brody and colleagues [10]. Combined, the existing literature and our report do not provide conclusive evidence that exposure to cigarette smoke, as defined by pack-years, affects brain morphometry. This is not completely unexpected due to the intrinsic confounds of human neuroimaging studies where subjective measures are used to draw associations between behavior and brain. Pack-years is a measure derived by the subjective responses of individuals to only two items regarding smoking history. The measure assumes that smoking behavior has not changed since initiation and that accurate reports can be provided. Although perfecting the pack-year measure will not abolish all of its inherent flaws, a better measure could and should be developed to examine the effects of cigarette smoke exposure on GMV.

As with any study conducted in drug-dependent individuals, inferences about what differences between groups in GMV signify cannot exclude the possibility that brain volume differed prior to cigarette smoking initiation. Pre-existing differences in GMV could predispose an individual to a number of aspects of drug use including experiencing increased positive responses to the reinforcing properties of the drug, reduced negative responses to its aversive properties, and/or reduced ability to inhibit aberrant behavior.

It should be noted that GMV differences between NDs and Controls in this and other studies could at least in part be a transient difference modulated by recent smoking. The use of T1-weighted scans to identify change in brain structure *in vivo* in humans has vulnerability: the T1 relaxation time for arterial blood and gray matter are not clearly distinguishable and therefore, apparent reported GMV findings might be at least partially related to changes in CBF or other physiological signals [58], [59]. For example, we observed medication-induced decreases in GMV in the cingulate and other regions that overlapped with changes in CBF following just one dose of medication [60]. If a single dose of medication can alter brain volume as indexed by VBM, it is conceivable that recent smoking could have a similar effect, especially in light of evidence that cigarette smoking induces regional alterations in CBF [41], [42], [61]. In the current study, NDs smoked a cigarette approximately 45 minutes prior to scan acquisition. Given that physiological responses to cigarette smoking dissipate within 20 min [plasma nicotine levels peak at 5 minutes and return to a steady concentration within 10 minutes [41] and myocardial blood flow measured after smoking returns to normal within 50 minutes [62], this should not be a confounding factor, but it cannot be ruled out.

This study is limited in that we did not obtain a full alcohol and/or illicit drug history on the subjects. However, having a significant alcohol and/or drug history was an exclusion criterion for both groups and urine drug screens verified the absence of illicit drugs in all subjects.

This study, conducted in a large cohort of NDs and matched Controls, contributes to our knowledge of brain morphology in nicotine addiction by providing in-depth analyses using an advanced registration technique and by expanding our knowledge on sex-specific effects in cigarette smokers. Identifying brain vulnerabilities with respect to sex provides a methodological framework for personalized therapies to improve relapse rates for both males and females.
